# CAR-T therapy for neuroendocrine neoplasms: a review of molecular targets and clinical translation

**DOI:** 10.3389/fimmu.2025.1695716

**Published:** 2025-10-22

**Authors:** Olga Golounina, Ildar Minniakhmetov, Rita Khusainova, Marina Loguinova, Ramil Salakhov, Hui Fan, Natalia Mokrysheva

**Affiliations:** ^1^ Department of Clinical Endocrinology, Endocrinology Research Centre, Moscow, Russia; ^2^ Laboratory of Genomic Medicine, Endocrinology Research Centre, Moscow, Russia; ^3^ Laboratory of Cell Biology, Endocrinology Research Centre, Moscow, Russia

**Keywords:** CAR-T therapy, neuroendocrine neoplasms (NENs), chimeric antigen receptor(CAR), somatostatin receptors (SSTR), DLL3, solid tumors, immunotherapy, personalized therapy

## Abstract

Neuroendocrine neoplasms (NENs) represent a heterogeneous group of malignancies. In metastatic and progressive forms, standard therapies are often ineffective. CAR-T therapy, which has demonstrated remarkable efficacy in hematologic malignancies, is considered a promising approach for NEN treatment, despite significant barriers inherent to solid tumors. This review aims to comprehensively analyze and systematize current scientific data on the development of CAR-T therapy for NENs of various origins. It focuses on relevant molecular targets, preclinical findings demonstrating the efficacy and safety profiles of CAR-T cells directed against potential targets, and the current status of early-phase (I/II) clinical trials for CAR-T therapy in NENs. The review examines major barriers to CAR-T therapy in solid tumors, including NENs, and presents innovative strategies to overcome them. Further research and clinical trials focusing on therapy personalization, enhanced safety and efficacy, and the development of combination approaches are essential for the successful integration of CAR-T therapy into clinical practice and for improving outcomes in patients with treatment-refractory NENs.

## Introduction

Neuroendocrine neoplasms (NENs) represent a heterogeneous group of relatively rare malignancies originating from cells of the diffuse neuroendocrine system. According to the unified World Health Organization (WHO) classification, all neuroendocrine neoplasms are designated by the term NEN, which encompasses well-differentiated neuroendocrine tumors (NETs) and poorly differentiated neuroendocrine carcinomas (NECs) (1).

NENs can arise in any organ containing neuroendocrine cells. However, the gastrointestinal tract and lungs represent the most frequent primary sites. Characterized by considerable clinical and morphological diversity and by their capacity to produce biologically active peptides and hormones, which frequently results in specific paraneoplastic syndromes, NENs remain a complex diagnostic and therapeutic challenge (2–4). For patients with advanced, metastatic, or progressive NENs, current treatment modalities frequently demonstrate suboptimal efficacy and are associated with significant adverse effects. This underscores the critical need for the active exploration and development of innovative, more effective, and personalized therapeutic strategies.

Within this context, immunotherapy, established over recent decades as one of the most dynamically evolving and promising fields in oncology, offers fundamentally novel mechanisms for combating tumors through the activation of the patient’s own immune system (5, 6). Among diverse immunotherapeutic approaches, significant interest lies in adoptive cell therapy utilizing genetically modified T-lymphocytes expressing chimeric antigen receptors (CAR-T therapy). Epitomizing the pinnacle of personalized medicine, CAR-T therapy integrates advances in molecular biology, immunology, and genetic engineering.

**Figure 1:**
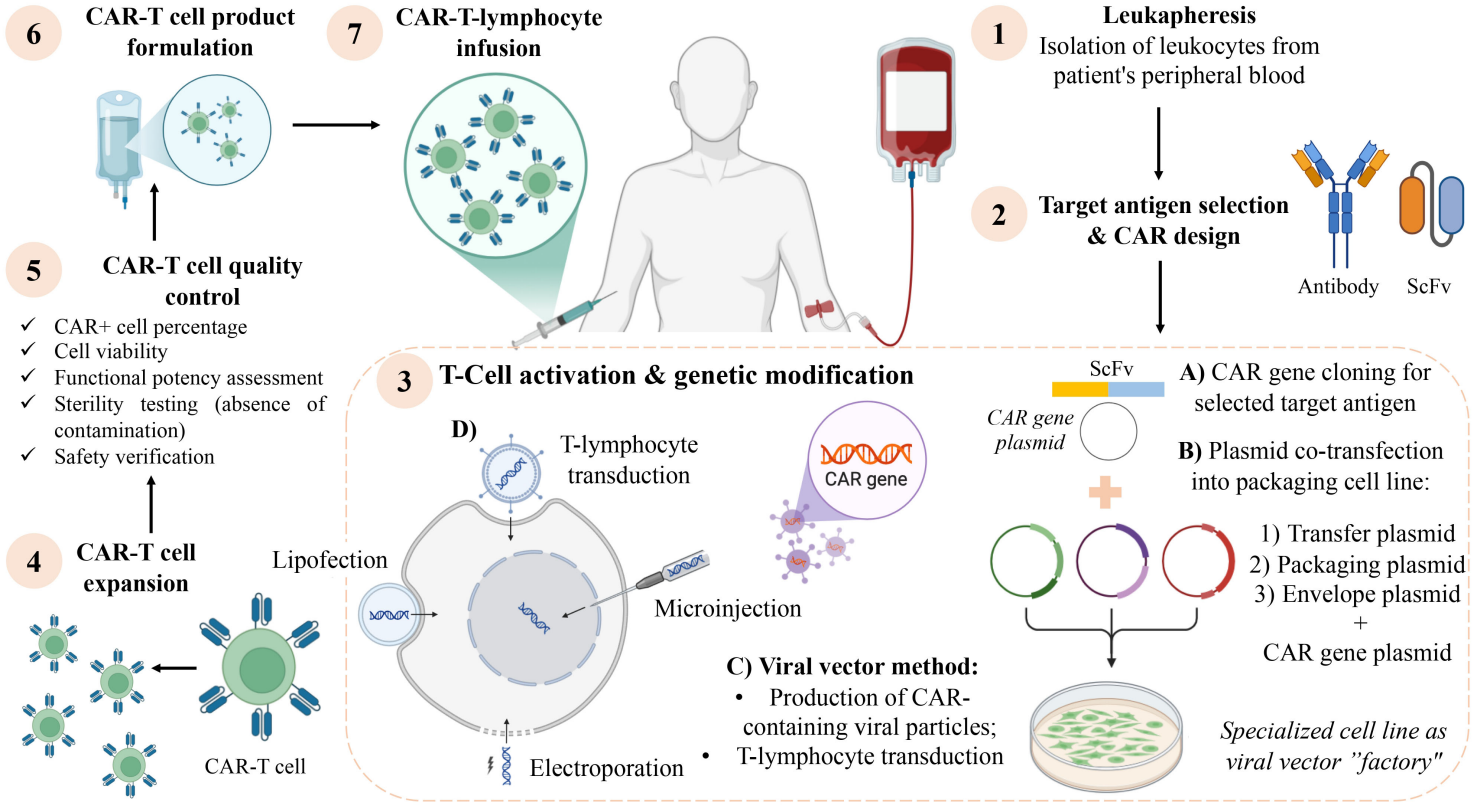
Flowchart illustrating the CAR-T cell therapy process. Steps include: 1) Leukapheresis for leukocyte isolation. 2) Target antigen selection and CAR design. 3) T-cell activation and genetic modification via lipofection, microinjection, or viral vectors. 4) CAR-T cell expansion. 5) CAR-T cell quality control, verifying sterility, viability, and potency. 6) CAR-T cell product formulation. 7) CAR-T lymphocyte infusion into the patient.

The principle of CAR T-cell therapy involves the *ex vivo* reprogramming of a patient’s autologous T-lymphocytes by equipping them with an artificially engineered receptor (CAR) designed to recognize a specific tumor-associated antigen. Following genetic modification and expansion, these cells are reinfused into the patient (**Figure 1**). While CAR-T therapy has demonstrated remarkable efficacy in hematologic malignancies such as multiple myeloma and relapsed/refractory B-cell lymphoproliferative disorders (leukemias, lymphomas) (7, 8), its application against solid tumors, including NENs, faces substantial challenges. Key barriers include the immunosuppressive tumor microenvironment (TME), which impedes T-cell infiltration and function through mechanisms such as immunosuppressive cytokine secretion (TGF-β, IL-10) and PD-L1 expression (9–11), and the frequent lack of ideal, homogeneously expressed tumor-specific antigens, increasing the risk of on-target, off-tumor toxicity.

To overcome the antigen selection hurdle, significant research focuses on identifying neoantigens and unique splice variants arising from genomic alterations and aberrant RNA splicing in tumors. The identification of tumor neoantigens is now feasible through advances in tumor DNA/RNA sequencing coupled with algorithms predicting peptide neoantigen affinity for patient MHC proteins. The study of alternative splicing offers an additional perspective. Pan-cancer analyses reveal approximately 30% more alternative splicing events in tumor tissues compared to normal tissues, providing a basis for discovering unique targets (12). Such discoveries have spurred interest in characterizing alternative splice variants as potential immunotherapy targets (13, 14). One promising approach involves identifying splice isoforms localized to the ectodomains, which may serve as tumor-specific antigens (15). The computational platform Isoform peptides from RNA splicing for Immunotherapy target Screening (IRIS) has been developed for their detection. IRIS analyzes long-read RNA sequencing (RNA-seq) data to identify unique splice variants expressed exclusively in tumors (16, 17). For instance, applying IRIS to solid tumor cell lines revealed previously unknown cancer-type-specific splice isoforms (15). Many of these isoforms are located on the ectodomains of proteins, which makes them accessible to CAR receptors, and their absence in healthy tissues significantly reduces the risk of on-target off-tumor toxicity (18). Thus, integrating long-read RNA sequencing, bioinformatic screening, and functional validation allows for personalized target selection for CAR-T therapy, which is particularly relevant for NENs, as rare and heterogeneous tumors where traditional antigens are often absent or poorly expressed.

The purpose of this review is to comprehensively analyze current scientific data on developing CAR-T therapy for NENs of various origins. It focuses on relevant molecular targets, preclinical findings, the status of early-phase clinical trials, and innovative strategies to overcome existing barriers, aiming to outline a path for the successful integration of CAR-T therapy into clinical practice for patients with refractory NENs.

## Methods

This comprehensive narrative review was conducted through a systematic literature search to identify relevant preclinical studies, clinical trials, and review articles on CAR-T therapy for neuroendocrine neoplasms. Electronic databases including PubMed/MEDLINE, Scopus, and Web of Science were queried for publications from inception through August 2025. Search strategies utilized a combination of keywords and Medical Subject Headings terms such as: “CAR-T”, “chimeric antigen receptor”, “neuroendocrine neoplasms”, “NEN”, “neuroendocrine tumor”, “NET”, “pancreatic neuroendocrine tumor”, “small cell lung cancer”, “SCLC”, “medullary thyroid carcinoma”, “MTC”, “DLL3”, “SSTR”, “CDH17”, “GFRα4”, along with specific NCT numbers of clinical trials. The reference lists of retrieved articles were also manually screened to identify additional relevant publications. Articles were selected based on their relevance to the review’s focus on molecular targets, preclinical efficacy, clinical translation, and innovative strategies for CAR-T therapy in NENs.

Given the narrative nature of this review and the focus on a rapidly evolving field, formal inclusion/exclusion criteria or a PRISMA flowchart were not employed; instead, the aim was to provide a comprehensive overview and synthesis of the current landscape. It is important to note that the clinical trials discussed are primarily in early phases (I/II), and no mature efficacy or toxicity results have been published in peer-reviewed literature yet, which limits systematic analysis of clinical outcomes.

## Advances in CAR-T therapy for pancreatic neuroendocrine neoplasms

Pancreatic NENs account for 2–5% of all pancreatic tumors and are classified as either functioning (associated with clinical syndromes caused by abnormal hormone production) or non-functioning neoplasms. Gastrointestinal and pancreatic neuroendocrine tumors (NETs) belong to the category of well-differentiated NENs and can have three malignancy grades (Grade 1 (G1), G2, G3). The G3 group comprises well-differentiated NETs of high malignancy grade, exhibiting high proliferative activity. Gastrointestinal and pancreatic neuroendocrine carcinomas (NECs) are poorly differentiated NENs of high malignancy grade and are subdivided into small-cell and large-cell NEC. Additionally, a category of mixed neuroendocrine-non-neuroendocrine neoplasms (MiNEN) is recognized (1, 19). In 10–20% of cases, pancreatic NENs may be associated with genetically determined hereditary syndromes, such as multiple endocrine neoplasia type 1 (MEN1), von Hippel-Lindau syndrome, neurofibromatosis type 1, tuberous sclerosis, and glucagon cell hyperplasia and neoplasia (20).

The heterogeneity of pancreatic NENs and the limited availability of known universal targets pose significant challenges for developing targeted therapies, including CAR-T. This drives the search for novel candidate antigens. To overcome the limitations of known tumor-associated antigens suitable for CAR-T therapy in NENs, Feng Z. et al. (21) developed a method for identifying new potential neoantigens suitable for targeting by CAR-T cells. The researchers employed phage display screening to identify single-domain antibodies (VHH) that selectively bind to gastrointestinal NEN cells. Recombinant analogs (derivatives) of VHH are also termed “nanobodies”, “single-domain antibodies”, or “mini-antibodies”. As a result, a VHH1 nanobody was isolated that specifically binds to the pancreatic neuroendocrine tumor cell line BON-1. VHH1 was identified as a specific target for cadherin-17 (CDH17), a membrane-bound adhesion protein overexpressed in gastrointestinal NEN cells (22, 23). *In vitro* and *in vivo* experiments (using fragments of mouse autochthonous tumors generated by *de novo* tumor initiation in normal cells within an intact organism) demonstrated that VHH1-based CAR-T cells (CDH17-CAR-T) are cytotoxic against both human and mouse tumor cells in a CDH17 expression-dependent manner. The researchers compared three CDH17-CAR-T constructs in three groups of mice transplanted with NT-3, a pancreatic islet NEN cell line expressing CDH17, leading to the development of CDH17+NT-3 tumors. Thirty-five days post-xenotransplantation, mice received CAR-T cell infusions, with treatment repeated five days later. The first group received CDH17-CAR-T with CD28 and 4-1BB co-stimulatory domains (VHH1-28BBz), the second group received CDH17-CAR-T lacking these co-stimulatory domains (VHH1-BBz), and the third group received unmodified T cells (CDH17-UTD). Results showed that VHH1-28BBz-CAR-T cells induced tumor mass reduction leading to complete tumor eradication after 42 days; VHH1-BBz-CAR-T cells demonstrated progressive volume reduction but failed to achieve complete tumor elimination, whereas CDH17-UTD therapy was ineffective and failed to control tumor growth. Tumors were also excised and analyzed ten days after the first infusion. Tissue analysis revealed an absence of neuroendocrine cells and an abundance of T-cells in tumors treated with VHH1-28BBz-CDH17-CAR-T cells, indicating rapid tumor elimination (21).

Chimeric Therapeutics, an Australian leader in cell therapy, announced on October 31, 2023, that the FDA had approved the Investigational New Drug (IND) application for CHM 2101 (CDH17-CAR-T), paving the way for further trials. The efficacy and safety of genetically modified autologous T-lymphocytes expressing CDH17 are currently being evaluated in the Phase I/II clinical trial NCT06055439.

Concurrently with the search for novel targets like CDH17, significant attention is directed towards somatostatin receptors (SSTRs), well-established targets in NEN diagnosis and therapy due to their selective expression on tumor cells. Based on the SSTR expression profile (24) and the proven efficacy of long-acting somatostatin analogs and radiolabeled somatostatin analogs in NEN treatment (25, 26), Mandriani B. et al. (27) developed CAR-T cells targeting SSTRs directly. The innovative CAR-T construct incorporated two molecules of octreotide, which binds with high affinity to SSTR2 and SSTR5, and the CD28 co-stimulatory molecule. CAR-T cells were transduced into CD8+ cells using a retroviral vector. CAR-T cell cytotoxicity was assessed *in vitro* on pancreatic NEN cell lines – BON1, QGP1, and CM – which overexpress SSTRs 1–5. After 72 hours of co-culture, tumor cell death was recorded at 58% (± 8%), 53% (± 1%), and 42% (± 3%), respectively. Once NEN xenografts reached a volume of 1 mm³, mice were randomized to receive intravenous injections of phosphate-buffered saline (PBS), unmodified T-cells, or anti-SSTR CAR-T cells. Tumor growth *in vivo* was assessed using bioluminescence. SSTR-targeted CAR-T cells significantly inhibited tumor growth in mouse models of subcutaneous NEN xenografts compared to control groups, with differences reaching statistical significance (p < 0.05) at day 14 and day 21 for CM and BON1 tumors, respectively. No adverse effects were observed in the animals during the 4 weeks post-treatment.

A key strategy for improving the safety of CAR-T therapy is the development of methods to control CAR-T cell activity, including the capability for on-off regulation. To eliminate CAR-T cells in case of severe adverse effects, mechanisms based on “suicide genes” have been developed. However, this leads to complete loss of the CAR-T cells (28, 29). The need for functional activity regulation led to the creation of adapter CAR-T cells (Ad-CAR T-cells). These cells recognize a label on a tumor antigen-binding molecule through the CAR receptor, forming a kind of activating “bridge” between the CAR and the target cell. Thus, the interaction between Ad-CAR-T cells and tumor cells depends on the presence of a bispecific adapter (30–34).

Applied to SSTR-targeted therapy for pancreatic NENs, a promising regulated approach has been proposed. Experimental data published in 2024 (35) suggest that a novel fluorescent SSTR2 peptide antagonist (Octo-Fluo) combined with anti-FITC adapter CAR (AdFITC(E2)-CAR) T-cells can function as a regulatable “bridge” for activation between the CAR and target cells expressing SSTR2. *In vitro* studies confirmed Octo-Fluo’s ability to bind to the Bon1-SSTR2 mCherry-Luc cell line (a model of pancreatic NEN) without signs of internalization. AdFITC(E2)-CAR-T cells were activated and effectively induced Bon1-SSTR2 cell death *in vitro*, depending on the concentration of Octo-Fluo. Similarly, *in vivo* studies in immunodeficient mice demonstrated that AdFITC(E2)-CAR-T cells combined with Octo-Fluo effectively infiltrated and eradicated Bon1-SSTR2 tumors. These processes also depended on the concentration of Octo-Fluo, with high doses saturating both the CAR receptor and the SSTR2 antigen independently, thereby inhibiting tumor infiltration and destruction. These results demonstrate the potential of using AdFITC(E2)-CAR-T cells with Octo-Fluo as a universal, regulatable adapter for targeted CAR-T cell immunotherapy of NENs with positive SSTR2 expression.

Thus, SSTRs, owing to their selective expression on the surface of neuroendocrine tumor cells and their involvement in key cellular functions, represent a unique target for developing highly effective and safe treatments, including CAR-T therapy. SSTRs, involved in regulating critical tumor cell functions such as suppressing hormone secretion, proliferation, and angiogenesis, form the basis for developing CAR-T cell therapies that could potentially be life-saving or even curative for patients with the most severe forms of the disease.

The approaches discussed – targeting CDH17, targeting SSTRs, and implementing adapter systems like Octo-Fluo/AdFITC – constitute promising directions for advancing CAR-T therapy for pancreatic NENs. The first key aspect involves the specific engineering of CAR-T cells recognizing CDH17 or SSTRs, enhancing treatment efficacy through precise tumor cell targeting with minimal damage to healthy tissues. The second important advantage stems from the use of regulatable approaches, such as adapter molecules (e.g., Octo-Fluo), which provide the means to control CAR-T cell activity, significantly reducing the risk of severe adverse effects. The third promising direction suggests the potential application of CAR-T therapy not only for advanced tumors but also at earlier disease stages, which could significantly increase the chances of successful cure. The fourth strategic opportunity lies in combining CAR-T therapy with conventional treatments, such as somatostatin analogs or targeted therapy, which could enhance the overall therapeutic effect and slow disease progression. Finally, further research and clinical trials, including the NCT06055439 trial for CDH17-CAR-T, will be crucial for refining safety and efficacy parameters and identifying patient populations who will derive the greatest benefit from this advanced technology.

The progress in pancreatic NENs underscores several themes relevant across NEN subtypes: the utility of established diagnostic targets (SSTRs) for therapy, the discovery of novel antigens like CDH17, and the critical importance of safety systems such as adapter CARs. However, the applicability of these targets varies. Unlike the prominent role of SSTRs in pancreatic and gastrointestinal NENs, lung NENs, particularly the most aggressive forms, have driven the exploration of a different set of antigens, with Delta-like ligand 3 (DLL3) emerging as a frontrunner.

## CAR-T therapy for lung neuroendocrine neoplasms: targeting DLL3 and alternative antigens

Lung NENs represent the second most common site for NENs after the gastrointestinal tract, accounting for 27.4% of all NENs across various localizations and up to 20% of all malignant lung tumors. The group of well-differentiated lung NENs includes typical and atypical carcinoids (NETs G1–G2). The group of poorly differentiated, high-grade lung NENs comprises neuroendocrine carcinomas (NECs), subdivided into large-cell NEC (LCNEC) and small-cell lung cancer (SCLC). SCLC and LCNEC are always high-grade malignancies and do not require additional numerical grade specification (36).

SCLC is an aggressive NEC subtype, constituting approximately 15% of all lung cancers. It is characterized by a high propensity for metastasis and an exceptionally malignant clinical course. Standard chemotherapy regimens for SCLC have largely exhausted their potential for improving therapeutic efficacy. The primary obstacle is the development of pronounced tumor resistance to chemotherapy (37). Unfortunately, the prognosis for SCLC patients remains poor, with a median overall survival of 10–12 months and 1- and 2-year overall survival rates of 56.2% and 21.7%, respectively (38).

The high prevalence of lung NENs, particularly aggressive forms like SCLC, coupled with their resistance to standard therapy, necessitates the exploration of novel approaches such as CAR-T therapy. The search for more personalized, innovative, and effective treatments for lung NENs, especially SCLC, represents not only a growing need in modern oncology but also a critical direction in advancing personalized medicine, which aims to tailor treatments to individual patient characteristics. CAR-T therapy is a promising avenue capable of transforming the treatment paradigm, particularly crucial for aggressive and therapeutically resistant cancers.

Delta-like ligand 3 (DLL3) stands as one of the most promising targets for CAR-T therapy in NENs across various localizations, including the lung. DLL3 is a non-canonical inhibitory ligand of Notch receptors and has been implicated in NEN pathogenesis (39–41). The Notch signaling pathway regulates most key cellular processes, which has stimulated increasing research interest in its role in various pathologies, including oncogenesis. Defects in this cascade are characteristic of multiple malignancies. Aberrant Notch signaling activity in most cancer types can exhibit both tumor-promoting and tumor-suppressive properties.

Notch signaling involves transmembrane receptors (Notch1–4 in humans) activated upon direct physical contact with cells expressing one of five ligands from the Jagged (JAG) or Delta-like (DLL) families (JAG-1, JAG-2, DLL1, DLL3, DLL4). Notch-1 regulates proliferation, invasion, and chemoresistance; Notch-2 is involved in tumor transformation initiation; Notch-3 regulates proliferation, cell migration, and chemoresistance; Notch-4 controls epithelial-mesenchymal transition and therapy resistance. Among the ligands, DLL1 regulates cell-cell communication, DLL3 inhibits apoptosis induction, DLL4 activates the NF-κB pathway, promoting VEGF expression; JAG-1 promotes angiogenesis, and JAG-2, upon binding Notch-2, activates tumor cell proliferation. Thus, Notch receptors and ligands are key players in processes that promote the malignant potential of tumors (42).

**Figure 2:**
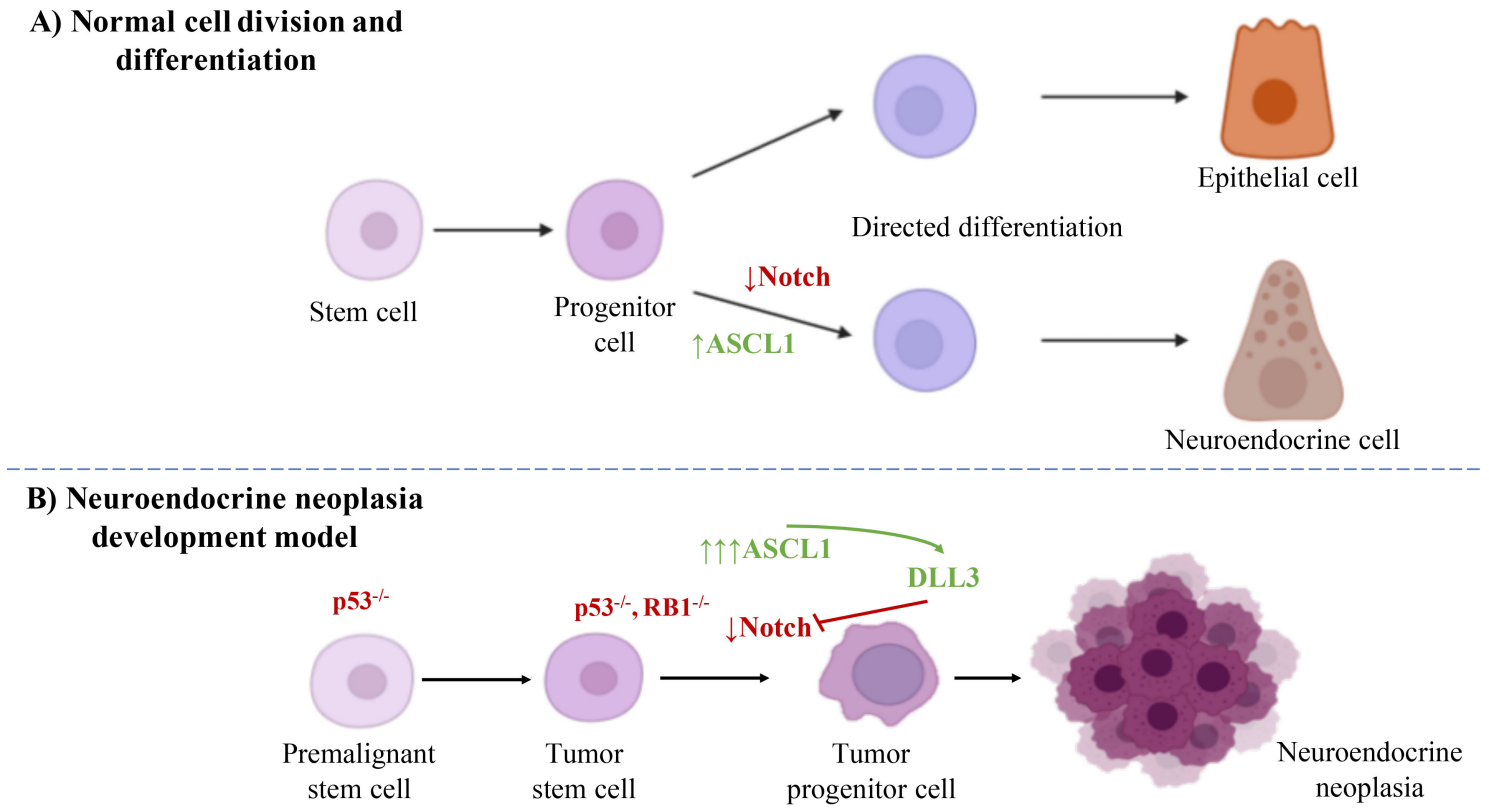
Illustration comparing normal cell development **(A)** and neuroendocrine neoplasia pathogenesis **(B)**, highlighting the role of DLL3. The model shows the evolution from a premalignant to a tumor progenitor cell, driven by ASCL1 upregulation and subsequent DLL3 expression. DLL3’s high expression in malignancies versus its low expression in normal tissues underscores its therapeutic promise [adapted from (43)].

DLL3 expression is regulated by the transcription factor ASCL1, which drives neuroendocrine cell differentiation and correlates with tumor-initiating potential (**Figure 2**) (43).

In normal tissues, DLL3 expression is typically low or absent. DLL3 expression is particularly elevated in SCLC and other NENs (44, 45). The first registered clinical trial (NCT03392064), a Phase I study, aimed to evaluate the safety and tolerability of AMG 119, an experimental DLL3-targeted CAR-T therapy, in patients with relapsed/refractory SCLC who had progressed after at least one platinum-based chemotherapy regimen. The study began on September 10, 2018, with a planned enrollment of 6 participants. However, it is currently listed as “Suspended” (prematurely terminated but may potentially resume). While the treatment was well-tolerated in the small cohort, the suspension may reflect challenges in achieving robust efficacy, a common hurdle in solid tumor CAR-T therapy, or strategic portfolio decisions by the sponsor.

Preliminary data from NCT03392064 were presented by Zhou D. et al. (46) in 2022 at the annual meeting of the American Society for Clinical Pharmacology and Therapeutics. The data indicated enrollment of five SCLC patients. All patients received AMG 119 at two dose levels: Cohort 1 (n=3) received 3 × 10^5^ CAR-T cells/kg; Cohort 2 (n=2) received 1 × 10^6^ CAR-T cells/kg. Both cohorts exhibited significant cellular expansion with prolonged persistence (up to 86 days). The treatment was well-tolerated. Three additional clinical trials are currently investigating DLL3-targeted CAR-T cells for lung NENs (**Table 1**).

**Table 1:** Registered clinical trials of anti-DLL3 CAR-T cells in lung NENs (ClinicalTrials.gov; data as of August 1, 2025).

Clinical trial	Year opened	Target	Indications	Phase	Status
NCT05680922	2023	DLL3 (LB2102)	SCLC, LCNEC	I	Recruiting
NCT06384482	2024	DLL3 (SNC115)	Relapsed/refractory SCLC or LCNEC	I	Recruiting
NCT06348797	2025	α-PD-L1/4-1BB DLL3 (BHP01)	Relapsed/refractory SCLC	I	Recruiting

Hermans BCM et al. (47) analyzed DLL3 expression in 94 stage IV LCNEC samples. Positive DLL3 expression (≥1% tumor cells) correlated with *STK11/KEAP1* mutations (characteristic of NSCLC-like LCNEC, as *STK11/LKB1* mutations are frequent in NSCLC). High DLL3 expression occurred in 74% of samples, including 100% of *STK11*-mutant and 91% of *KEAP1*-mutant cases, highlighting DLL3’s therapeutic potential. Higher DLL3 expression was observed in tumors expressing ≥2 neuroendocrine markers (chromogranin A, synaptophysin, CD56) compared to those expressing only one marker (81% [66/82] vs. 33% [3/9]). However, causality and therapeutic applicability require further investigation.

Ogawa H et al. (48) examined the relationship between DLL3 expression and efficacy of platinum-based adjuvant chemotherapy. In DLL3-positive LCNEC patients (n=23/70, 32.9%), adjuvant chemotherapy showed no significant difference in 5-year overall survival (58.3% vs. 35.7%; p=0.360) or disease-free survival (41.7% vs. 35.7%; p=0.740) versus no adjuvant therapy. Conversely, DLL3-negative patients receiving adjuvant chemotherapy showed statistically significant improvements in 5-year OS (90% vs. 26.9%; p<0.01) and disease-free survival (80% vs. 21.7%; p<0.01). These results suggest that DLL3 may predict prognosis and chemosensitivity in LCNEC. Integrating DLL3 expression with histopathological, immunohistochemical, and molecular profiling could optimize personalized adjuvant therapy decisions, potentially improving long-term survival outcomes.

Recent preliminary data from the phase I trial of LB2102 (NCT05680922) have provided the first clinical evidence of activity for DLL3-targeted CAR-T therapy in lung NENs. In 12 patients with relapsed/refractory SCLC and LCNEC across four dose levels (0.3-4.0 × 10^6^ CAR-T cells/kg), LB2102 demonstrated early antitumor activity with an objective response rate of 16.7% and a disease control rate of 66.7%. Importantly, no dose-limiting toxicities were observed through the highest tested dose level, and the safety profile was manageable with only grade 1 cytokine release syndrome occurring in two patients and no neurotoxicity reported. CAR-T cell expansion showed dose-dependent kinetics, with responses deepening at higher dose levels. These encouraging early results support continued dose escalation and further development of LB2102 (49).

Concurrently, several other DLL3-targeted CAR-T approaches are under investigation in early-phase trials for lung NENs, including SNC-115 (NCT06384482) and BHP01, a novel construct incorporating α-PD-L1/4-1BB signaling domains (NCT06348797). Additionally, preclinical development of allogeneic DLL3 CAR-T candidates (ALLO-213) is underway (50).

### DLL3 expression in other neoplasms

DLL3 expression has been investigated across diverse neoplasms, revealing distinct biological and clinical patterns. Ingenwerth M. et al. (51) analyzed 59 formalin-fixed paraffin-embedded (FFPE) samples of medullary thyroid carcinoma (MTC) with and without stromal desmoplasia. Tumors exhibiting desmoplasia and lymph node metastasis (≥50%) demonstrated high DLL3 expression, whereas non-desmoplastic specimens showed low DLL3 levels. This suggests DLL3 may identify more aggressive neoplasms with greater propensity for early metastasis.

In another study, Cimic A. et al. (52) examined 62 cervical NECs and 599 cervical squamous cell carcinomas. DLL3 was detected in 81% of NECs, with diffuse expression (≥50% tumor cells) observed in 49% of cases. Expression intensity was higher in metastatic lesions than primary tumors and showed an inverse correlation with prevalent pathogenic mutations in *PIK3CA* (17%) and *PTEN* (10%). Notably, DLL3 was the sole overexpressed biomarker identified in this cohort.

DLL3 expression has also been found in gastrointestinal mixed adenoneuroendocrine carcinomas, particularly in tumor cells with positive immunoreactivity to chromogranin A (53). Liverani C. et al. (54) studied the diagnostic and prognostic role of DLL3 in 47 gastroenteropancreatic neuroendocrine neoplasms (GEP-NENs). DLL3 was undetectable in well-differentiated GEP-NETs but expressed in 76.9% of poorly differentiated neoplasms. It is important to note that DLL3 expression was correlated with significantly reduced progression-free survival and overall survival.

High DLL3 expression is characteristic of neuroendocrine prostate cancer – an aggressive variant of castration-resistant prostate cancer with poor prognosis. Puca L. et al. (55) detected DLL3 expression in 76.6% (36/47) of samples, with its presence correlating significantly with aggressive clinical course, worse overall survival (35 vs. 81.5 months), and shorter time to metastasis development (11.8 vs. 71.6 months).

Xie H. et al. (56) conducted immunohistochemical analysis of DLL3 in 65 Merkel cell carcinoma samples, a malignant non-melanocytic neuroendocrine skin tumor. Positive DLL3 expression was observed in 89% (58/65) of cases, but this finding does not seem to have prognostic significance due to the lack of correlation with overall survival.

**Figure 3:**
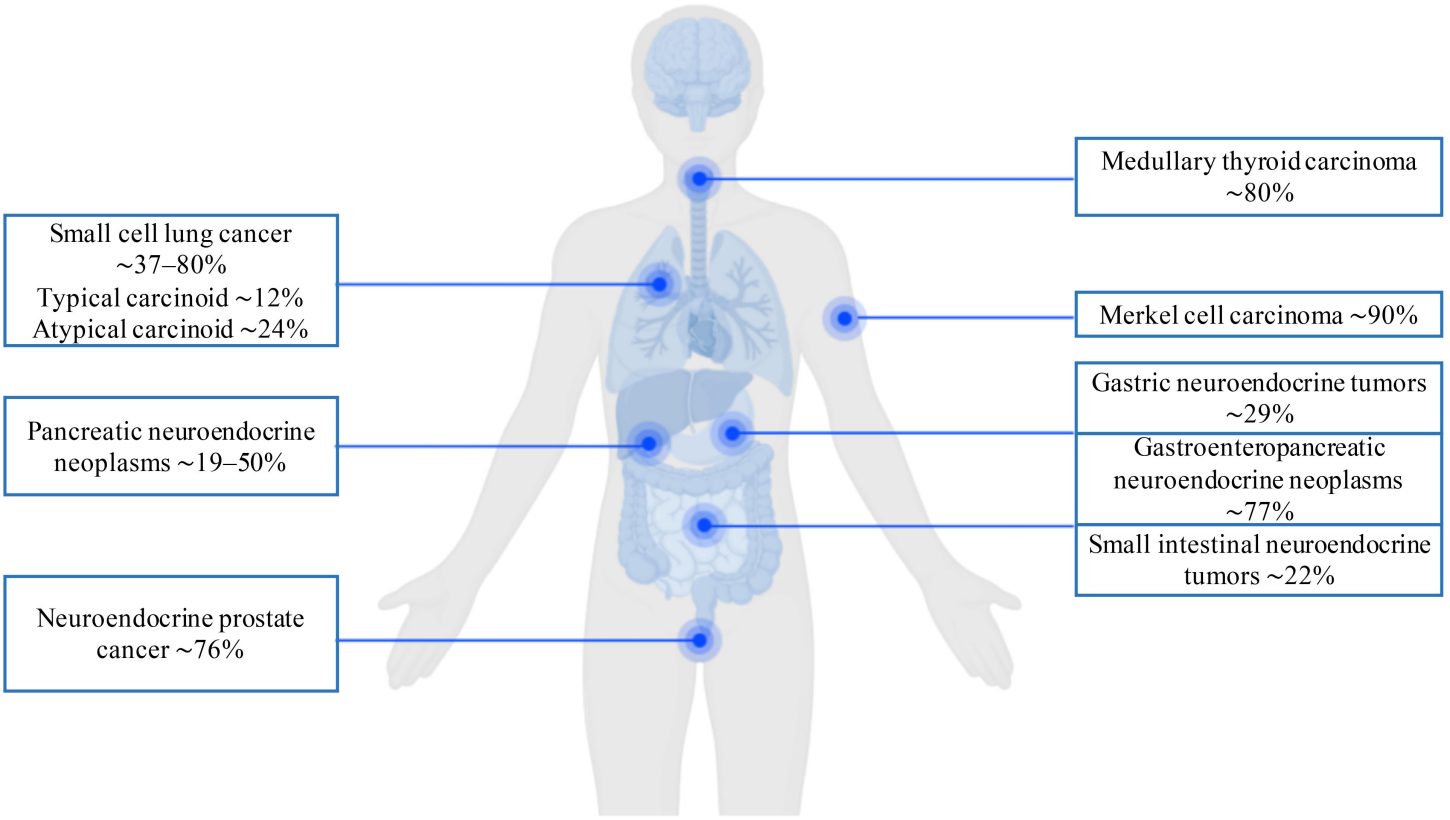
Immunohistochemical prevalence of DLL3 expression in neuroendocrine neoplasms.

The summarized prevalence of DLL3 expression across NENs, as determined by immunohistochemical analysis, is presented in **Figure 3**. These data demonstrate that elevated DLL3 expression characterizes not only pulmonary NENs but also diverse aggressive malignancies beyond the lung. Given its frequent overexpression across the wide spectrum of neoplasms, DLL3 represents a compelling therapeutic target for CAR-T therapy in refractory tumors, including lung NENs.

### Beyond DLL3: emerging molecular targets for CAR-T therapy in lung neuroendocrine neoplasms

While DLL3 remains the most extensively studied target for CAR-T therapy in lung NENs, several other promising antigens are under investigation. Less explored yet potentially significant molecular targets for CAR-T therapy in pulmonary NENs include disialoganglioside (GD2), neural cell adhesion molecule 1 (NCAM-1/CD56), and B7 homolog 3 (B7-H3/CD276).

### GD2: a pan-cancer target with therapeutic challenges

GD2 is a surface antigen expressed across a broad spectrum of neuroectodermal and epithelial tumors, including neuroblastoma, melanoma, glioma, retinoblastoma, medulloblastoma, SCLC, breast cancer, sarcoma, bladder cancer, colorectal cancer, and prostate cancer. Physiologically, GD2 expression occurs in normal cells of the central and peripheral nervous systems, melanocytes, lymphocytes, dendritic cells, and mesenchymal stem cells. Gangliosides participate in cellular interaction, adhesion, proliferation, and differentiation (57). However, GD2 expression is significantly elevated in malignant cells, rendering it suitable not only for therapeutic targeting but also for diagnostic and prognostic applications (58).

GD2 expression is documented across diverse malignancies, with the spectrum of GD2-positive tumors continually expanding. Detectable in circulating blood, GD2 concentrations correlate with tumor stage (59). The antigen exhibits genetic stability: its expression persists during treatment, and most surface-bound GD2 remains accessible after antibody binding and immune recognition (60). This property makes GD2 a promising target for immune therapy. However, GD2-targeted therapies face limitations due to toxicity concerns and suboptimal efficacy in advanced solid tumors.

A recent study by Reppel L. et al. (61) investigated GD2-targeted CAR-T cells (GD2-CAR-T) against SCLC cell lines and*in vivo* models of primary/metastatic SCLC. GD2 was overexpressed in SCLC cells, validating its therapeutic potential. To enhance CAR-T expansion and persistence, the GD2-CAR construct incorporated IL-15 and utilized an scFv derived from monoclonal antibody 14G2a. A GD2.CAR.15 cassette was engineered using a 2A peptide sequence to co-express the optimized GD2-CAR with IL-15 (62). *In vitro*, GD2-CAR-T effectively lysed GD2+ cells. In orthotopic xenograft models, these cells demonstrated potent antitumor activity against GD2+ SCLC tumors. Notably, combining the EZH2 inhibitor tazemetostat enhanced GD2 expression and increased tumor sensitivity to GD2-CAR-T cytotoxicity.

### CD56 (NCAM-1): HLA-independent targeting in neuroendocrine malignancies

Another CAR-T approach, developed by Crossland DL. et al. (63), targets CD56 (NCAM-1) – a glycoprotein highly expressed on malignant cells of neuronal/neuroendocrine origin, including SCLC (independent of HLA expression). CD56-CAR-T exhibited significant cytolytic activity against CD56+ SCLC cell lines and other malignancies *in vitro* and *in vivo.* CD56 has previously been targeted by antibody-based therapies with proven preclinical efficacy (64). The study demonstrated that CD56-CAR-T specifically lysed CD56+ SCLC cells and other CD56+ malignancies, achieving up to 64.9% target cell lysis. In SCLC xenograft models, CD56 targeting resulted in substantial tumor reduction by day 20 post-injection and improved overall survival, suggesting CD56-CAR-T as a viable immunotherapeutic strategy for CD56+ SCLC (63).

### B7-H3 (CD276): an immune modulator with dual roles

B7 homolog 3 (B7-H3/CD276), a type I transmembrane glycoprotein, is overexpressed in tumor tissues (including SCLC) but shows limited distribution in normal tissues (65). Its physiological function remains debated, with evidence supporting both co-stimulatory and co-inhibitory roles in T-cell response regulation. Additionally, B7-H3 promotes tumor migration, invasion, progression, metastasis, and therapy resistance (66).

B7-H3 represents both a prognostic marker and therapeutic target. While specific data on B7-H3-directed CAR-T for lung NENs are lacking, clinical trials targeting B7-H3 in other solid tumors support its promise. Further studies are needed to evaluate B7-H3 expression in pulmonary NENs and develop corresponding CAR-T constructs.

The extensive investigation of DLL3 in lung NENs highlights a target with broad expression across high-grade NENs of various origins, yet its expression is often low or absent in well-differentiated counterparts. This pattern of target expression dependent on tumor grade and differentiation is a recurring theme. In contrast, medullary thyroid carcinoma (MTC), a well-differentiated NEN, has prompted the identification of a highly specific target, GFRα4, demonstrating the need for a tailored approach based on the unique molecular landscape of each NEN subtype.

## CAR-T therapy for medullary thyroid carcinoma: from preclinical development to GFRα4-CAR clinical trials

Medullary thyroid carcinoma (MTC) is a malignant tumor arising from parafollicular neuroendocrine cells (C-cells) of the thyroid gland that produce calcitonin. It accounts for 3–5% of all thyroid malignancies (67, 68). In 20–25% of cases, MTC occurs as part of multiple endocrine neoplasia syndromes type 2A or 2B (MEN2A/MEN2B), while the remainder are sporadic (69, 70). The clinical course of MTC is highly variable and unpredictable, ranging from indolent to highly aggressive, depending on multiple factors. Despite improved detection in recent years, most patients are diagnosed at stages III–IV (71, 72).

Systemic chemotherapy demonstrates limited efficacy in metastatic MTC, achieving clinical remission in ≤ 30% of cases, typically of short duration. Long-acting somatostatin analogs and the mTOR inhibitor everolimus have shown promise as therapeutic options (73). Two oral tyrosine kinase inhibitors, vandetanib and cabozantinib, are FDA- and EMA-approved for progressive MTC (74). While they improve progression-free survival, no overall survival benefit has been demonstrated. Importantly, these agents are non-selective, and toxicity-related adverse events are substantial (75). Recent years have seen the emergence of highly selective RET kinase inhibitors (e.g., pralsetinib/BLU-667, selpercatinib/LOXO-292) with efficacy in MTC (76). However, their use requires testing for germline and somatic *RET* mutations (77).

Given these limitations, CAR-T cell immunotherapy represents a promising approach for MTC. Based on GFRα4 expression in MTC cells, Bhoj VG. et al. (78) hypothesized GFRα4 as a potential CAR-T target. GFRα4, a member of the glial cell line-derived neurotrophic factor (GDNF) receptor alpha family, is expressed in MTC cells. Its specific binding to the GDNF-family ligand persephin (PSPN) induces RET phosphorylation, activating downstream signaling pathways and triggering physiological effects (79).

Using phage display, the researchers engineered P4-10bbz – a GFRα4-targeted CAR construct. This CAR was cloned into a lentiviral vector, and anti-GFRα4 CAR-T cells were generated using viral transduction. *In vitro* studies demonstrated P4-10bbz CAR specificity for GFRα4. To assess CAR activation, P4-10bbz was expressed in Jurkat cells containing an NFAT-activated green fluorescent protein reporter. Flow cytometry confirmed activation of P4-10bbz-expressing Jurkat cells upon exposure to recombinant/soluble GFRα4 or co-culture with GFRα4+ human MTC cell lines (TT, MZ-CRC1). Primary human T-cells transduced with P4-10bbz lysed 60–70% of TT and MZ-CRC1 cells in co-culture. Elevated IL-2 and IFNγ levels in conditioned media confirmed CAR-T activation upon engagement with GFRα4+ targets. *In vivo* efficacy was demonstrated in murine xenograft models of MTC, where bioluminescent imaging revealed a significant reduction in tumor burden following CAR-T cell administration. Expansion of circulating CAR-T cells correlated with antitumor response, evidencing CAR-T functionality (78).

Currently, GFRα4-CAR-T cells are being evaluated in a Phase I clinical trial (NCT04877613) for adults with recurrent/metastatic MTC who received ≥ 1 prior tyrosine kinase inhibitor regimen or declined such therapy. Patients underwent lymphodepletion with fludarabine/cyclophosphamide. The primary endpoint, consistent with most Phase I studies, is the assessment of safety and toxicity profiles, which encompasses the incidence and severity of treatment-related adverse events graded according to CTCAE v5.0. Secondary endpoints include duration of response, overall survival, and relapse-free survival. The trial started on August 19, 2021, with a planned enrollment of 18 participants. Recruitment is ongoing, with an estimated completion date of June 1, 2039 NCT04877613.

While GFRα4-directed CAR-T therapy is presently the lead investigational approach for MTC, **Table 2** details additional targets under clinical evaluation in non-medullary thyroid carcinomas.

**Table 2:** Registered clinical trials of CAR-T therapy for thyroid cancer (ClinicalTrials.gov; data as of August 1, 2025).

Clinical trial	Year opened	Target	Indications	Phase	Status
NCT04119024	2019	IL13Ralpha2	Recurrent/Refractory Metastatic Thyroid Cancer	I	Recruiting
NCT04420754	2020	ICAM-1	Recurrent/Refractory Poorly Differentiated Thyroid Carcinoma, Anaplastic Thyroid Cancer	I	Active, not recruiting
NCT04877613	2021	GFRα4	Recurrent or Metastatic Medullary Thyroid Carcinoma	I	Active, not recruiting

The development of GFRα4-specific CAR-T cells represents a significant step in advancing therapeutic options for MTC patients, especially given the limitations of existing therapies. The ongoing Phase I clinical trial (NCT04877613 https://clinicaltrials.gov/study/NCT04877613) constitutes the first CAR-T study specifically designed for MTC, reflecting growing interest in immunotherapeutic approaches for this malignancy. GFRα4-directed CAR-T therapy may emerge as a viable therapeutic option, potentially complementing or surpassing the efficacy of existing targeted agents for select patient subgroups. However, realizing the full potential of this approach requires not only data from the current trial but also subsequent Phase II/III studies focused on optimizing treatment protocols, managing toxicity profiles, and identifying predictors of response.

## Systematic evaluation of clinical trial landscape of CAR-T cell therapy for NENs and future directions

The clinical development of CAR-T therapy for NENs is in early stages, with all current trials in Phase I or I/II. Common inclusion criteria across trials include progression after standard therapy (e.g., platinum-based chemotherapy for SCLC, tyrosine kinase inhibitors for MTC) and confirmation of target antigen expression.

Trial designs primarily focus on safety endpoints (dose-limiting toxicities, adverse events) and determination of maximum tolerated dose or recommended Phase II dose. Efficacy endpoints typically include objective response rate by RECIST criteria and disease control rate (**Table 3**).

**Table 3:** Registered clinical trials of CAR-T cell therapy for neuroendocrine neoplasms.

Clinical trial	Target	Phase	Indications	Key inclusion criteria	Primary outcome measures	Preliminary results
NCT06055439	CDH17	I/II	Advanced GEP-NETs (relapsed or refractory)	CDH17+ tumors, progressed on prior therapy	Dose-limiting toxicity, objective response rate, rates and grades of cytokine release syndrome, all other adverse events and toxicities	No results posted (trial ongoing)
NCT04877613	GFRα4	I	Recurrent/metastatic medullary thyroid carcinoma	≥1 prior tyrosine kinase inhibitor or declined tyrosine kinase inhibitor	Incidence of treatment-emergent adverse events as assessed by CTCAE v5.0.	No results available
NCT05680922	DLL3 (LB2102)	I	Relapsed/refractory SCLC or LCNEC	Progressed after platinum therapy	Safety and tolerability of LB2102, recommended dose for expansion, maximum tolerated dose or recommended Phase II dose	Objective response rate 16.7%, disease control rate 66.7% (n=12); no dose-limiting toxicities through dose level 4 (4.0 × 10^6^ CAR-T cells/kg); manageable safety profile (49)
NCT06384482	DLL3 (SNC115)	I	Relapsed/refractory SCLC or LCNEC	Progressed after ≥1 prior line	Safety of SNC115 injection	No results available
NCT06348797	DLL3 (BHP01)	I	Relapsed/refractory SCLC or LCNEC	Progressed after platinum therapy	Dose-limiting toxicity	No results available
NCT03392064	DLL3 (AMG119)	I	Relapsed/refractory SCLC or LCNEC	Progressed after platinum therapy	Safety, tolerability	Well-tolerated in small cohort (n=5); suspended potentially due to efficacy challenges
NCT04119024	IL13Ralpha2	I	Recurrent/metastatic Thyroid Cancer	Refractory to standard therapy	Dose-limiting toxicity, incidence of adverse events	No results available
NCT04420754	ICAM-1	I	Poorly differentiated/Anaplastic thyroid cancer	Unresectable/metastatic disease	Incidence of overall Grade ≥3 adverse events and serious adverse events, incidence of anticipated AIC100 CAR-T cell related adverse events, serious adverse events and adverse events of special interest, recommended Phase II dose	No results available

The suspension of the AMG 119 (DLL3-CAR) trial (NCT03392064) after limited enrollment highlights translational challenges in solid tumor CAR-T therapy, potentially reflecting difficulties in achieving robust efficacy or strategic portfolio decisions. In contrast, preliminary data from the LB2102 trial (NCT05680922) demonstrate early signals of activity with manageable safety, supporting continued dose escalation.

Several limitations characterize the current clinical landscape: small patient cohorts, focus on heavily pretreated populations, and heterogeneity in trial design and endpoints that limits cross-trial comparisons. The absence of mature, peer-reviewed results from most trials underscores the nascent stage of this field.

Despite the considerable optimism fueled by preclinical successes and the initiation of clinical trials, it is crucial to maintain a balanced perspective regarding CAR-T therapy for NENs. The current clinical landscape is marked not only by encouraging preliminary results but also by significant setbacks. The suspension of trials like the DLL3-targeting AMG 119 (NCT03392064) underscores the formidable challenges in achieving robust efficacy in solid tumors, potentially relating to insufficient T-cell persistence, a hostile TME, or antigen heterogeneity. Acknowledging these failures is essential for guiding future research and setting realistic clinical expectations.

Furthermore, while CAR-T therapy is a quintessential example of personalized medicine, it is not the only immunotherapeutic modality under investigation. Competing strategies such as bispecific T-cell engagers (e.g., DLL3-targeting agents), antibody-drug conjugates, and cancer vaccines offer alternative mechanisms for engaging the immune system, often with less complex manufacturing and more manageable toxicity profiles. The exploration of alternative effector cells, like CAR-NK cells, is also gaining traction due to their potential for “off-the-shelf” allogeneic use and a more favorable safety profile, potentially overcoming key limitations of autologous CAR-T products.

Finally, the profound practical challenges of cost, manufacturing complexity, and scalability must be critically examined for successful real-world translation. The personalized, autologous nature of current CAR-T therapies renders them exceptionally resource-intensive, limiting access and posing significant economic burdens on healthcare systems. Widespread adoption for NENs will be contingent not only on demonstrating efficacy but also on developing more cost-effective, scalable manufacturing processes and sustainable reimbursement models.

## Overcoming barriers in CAR-T therapy for neuroendocrine neoplasms

CAR-T therapy faces significant challenges in solid tumors like NENs, which can be categorized into three primary areas: 1) the immunosuppressive and physically obstructive TME, 2) the scarcity of safe and homogeneously expressed tumor-specific antigens, and 3) practical limitations regarding toxicity, cost, and complex manufacturing. A multifaceted research effort is underway to address these hurdles, focusing on enhancing CAR-T cell intrinsic properties, modifying the TME, and developing rational combination strategies.

Substantial efforts focus on optimizing CAR design to improve persistence and efficacy. This includes engineering novel costimulatory domains (4-1BB/CD137, CD28, ICOS/CD278) and signaling components (e.g., CD40/MyD88, DAP10/12). For example, third-generation CARs combining ICOS (CD278) and 4-1BB (CD137) demonstrated superior antitumor efficacy in lung tumors with prolonged *in vivo* persistence (80). Phase I clinical trial further support CAR-T cells targeting TnMUC1 (Mucin 1, also known as episialin, PEM, H23Ag, EMA, CA15-3, or MCA) with dual CD2/CD3ζ signaling domains in metastatic solid tumors (81). Additional approaches include integrating CD40 with TLR-adapter MyD88 to improve persistence and efficacy (82, 83), and incorporating DNAX-activating proteins (DAP10/12) for enhanced antitumor activity (84, 85). Nevertheless, no consensus exists regarding optimal costimulatory domain combinations for solid tumors.

Other developments include safety-enhanced CARs with logic-gated activation controls, multispecific CARs targeting multiple antigens, and localized delivery systems to reduce systemic toxicity. Research also explores strategies to counteract the immunosuppressive TME, focusing on engineering TME-resistant CAR-T cells and developing adjunct therapies targeting TME factors to amplify efficacy. Alternative immune effector platforms, such as CAR-NK and CAR-macrophage (CAR-M) cells, offer complementary mechanisms that may augment or replace conventional CAR-T approaches in specific clinical contexts (86, 87).

Another significant area of focus is combination therapy. The use of CAR-T cells alongside immune checkpoint inhibitors (anti-PD-1/PD-L1) is viewed as a promising strategy to overcome the immunosuppressive influence of the NEN tumor microenvironment (88). Unlike many hematological malignancies, which often lack local immune suppression mechanisms, solid tumors are significantly infiltrated by diverse cell types that promote their growth, angiogenesis, and metastasis. Among the most prominent immunosuppressive cells within the tumor microenvironment are regulatory T-cells (Tregs), myeloid-derived suppressor cells (MDSCs), and tumor-associated macrophages (TAMs), all of which limit CAR-T cell efficacy. The application of antibodies targeting the activity of these cells, combined with genetic engineering approaches designed to deplete Tregs and MDSCs, has demonstrated enhanced efficacy of CAR-T cell therapy in preclinical animal studies (89).

Clinical data, for instance in pleural mesothelioma, has shown the potential of such combinations to increase median overall survival. A Phase I study evaluated a strategy enhancing CAR-T cell therapy (autologous mesothelin-targeted CAR-T cells) by combining it with the PD-1 checkpoint inhibitor pembrolizumab. The median overall survival reached 23.9 months, with a partial response according to mRECIST (modified Response Evaluation Criteria in Solid Tumors) criteria observed in 12.5% (n=2/16) of cases, stable disease in 56.3% (n=9/16), and disease progression in 31.3% (n=5/16). The median time to initiation of pembrolizumab therapy following CAR-T cell infusion was 6 weeks (range 4 to 17 weeks) (90). Combining CAR-T therapy with immune checkpoint inhibitors thus appears a potential strategy for enhancing CAR-T cell efficacy and requires further investigation (91).

Alternative approaches involve engineering CAR-T cells to secrete anti-PD-L1 antibodies or utilizing knockout technologies (CRISPR/Cas9) to eliminate inhibitory molecules (PD-1, CTLA-4, LAG-3) within the CAR-T cells themselves (92–96).

Substantial efforts are directed towards modifying the TME and improving CAR-T cell infiltration. To combat immunosuppression, CAR-T cells can be engineered to secrete immunostimulatory cytokines (IL-12, IL-15, IL-18, IL-23) that modulate the TME (97–99). Overcoming physical barriers, such as the extracellular matrix, remains a key challenge. Relevant strategies include local delivery of CAR-T cells, developing methods for matrix disruption, and engineering solutions like the triple knockdown of key adhesion/migration molecules (CD11a, CD49d, PSGL1). This approach selectively restricts migration to healthy tissues while preserving antitumor activity, as demonstrated in models using anti-EpCAM CAR-T cells (100). At the same time, CAR-T cells capable of expressing receptors for specific chemokines present in the tumor microenvironment are under development. To enable targeted trafficking of CAR-T cells into tumors, cells expressing receptors for chemokines specifically produced within the tumor microenvironment (e.g., CXCR2/CXCL1, CCR2b/CCL2, CXCR1 (2)/IL-8(CXCL8), CX3CR1/CX3CL1, CSF-1R/CSF-1) are being engineered. Preclinical studies confirm that such modifications significantly enhance tumor infiltration and antitumor efficacy (101–104). In an *in vivo* study, CAR-T cells expressing CX3CR1 demonstrated enhanced migration towards tumor cells producing CX3CL1, leading to tumor regression (105). In another preclinical study, Lo AS et al. (106) engineered CAR-T cells expressing the receptor for macrophage colony-stimulating factor-1 (CSF-1R), which binds to CSF1, a chemokine that recruits monocytes and is synthesized by tumor cells, thereby enhancing CAR-T cell infiltration. These research findings collectively demonstrate how various modifications to CAR-T cells can enhance their antitumor effect.

A distinct research direction focuses on developing multispecific CAR platforms aimed at increasing specificity, overcoming antigen heterogeneity, and reducing the risk of tumor escape. Such platforms include: Dual CAR, expressing two independent receptors and requiring simultaneous binding of both antigens for activation (high specificity) (107); TanCAR (tandem CAR), incorporating two antigen-binding domains within a single receptor and providing synergistic activation upon binding either antigen (lower specificity) (108); and iCAR (inhibitory CAR), which suppresses activation of the primary CAR upon binding an antigen expressed on normal cells, thereby reducing off-target toxicity (109).

Therefore, successfully overcoming the barriers to CAR-T therapy for NENs necessitates a comprehensive approach integrating advances in genetic engineering, targeted modification of the TME, and the development of rational combination regimens. This multimodal strategy is essential for the successful integration of CAR-T therapy into the clinical management of resistant and metastatic NENs.

## Conclusion

NENs represent a heterogeneous group of malignancies with limited therapeutic options for patients with metastatic or progressive disease. CAR-T therapy, which has demonstrated high efficacy in hemato-oncology, is regarded as a promising approach for treating NEN, despite significant barriers inherent to solid tumors. Successful preclinical studies and the initiation of the first clinical trials (NCT06055439 targeting CDH17, NCT04877613 targeting GFRα4, NCT05680922, NCT06384482, NCT06348797 targeting DLL3) represent critical advances.

An integrated view across NEN subtypes reveals both common challenges and unique opportunities. Pancreatic NENs are at the forefront with targets like CDH17 and SSTRs, the latter offering innovative regulatable adapter systems (e.g., Octo-Fluo/AdFITC). Lung NENs, particularly SCLC, have seen the most clinical activity focused on DLL3, though early trials highlight the need to overcome antigen heterogeneity and immunosuppression. For MTC, GFRα4 represents a highly specific target moving through clinical validation.

It is also crucial to acknowledge the spectrum of NENs beyond these common types. Pheochromocytomas and paragangliomas (PPGLs) present a unique challenge, characterized by a T-cell depleted and macrophage-dominated tumor microenvironment, which may limit the efficacy of current CAR-T approaches designed for more immunogenic tumors. While early-phase trials exploring immune checkpoint inhibitors like Penpulimab (NCT05885399) are underway, the development of CAR-T for PPGLs is hindered by a lack of widely expressed, safe target antigens and the need to overcome a profoundly immunosuppressive TME. Future efforts must focus on target discovery and TME reprogramming strategies for these malignancies (3, 4).

A practical framework for future development must prioritize several key areas, beginning with target discovery and personalization through comprehensive genomic and transcriptomic profiling to identify ideal targets on a patient-specific basis. It must also focus on CAR construct optimization to enhance efficacy and safety, alongside the development of rational combination strategies with other therapies to overcome resistance mechanisms. Despite this potential, significant hurdles such as high costs, complex manufacturing, and the management of acute toxicities remain barriers to widespread adoption.

For CAR-T therapy to move beyond a one-size-fits-all approach and become a mainstream option for NENs, a robust practical framework for personalization must be established. This extends beyond simply identifying a target to a comprehensive, biomarker-driven strategy. A proposed pathway first requires comprehensive profiling through mandatory deep molecular characterization of tumor biopsies at progression to identify not only overexpressed antigens but also neoantigens and tumor-specific splice variants. A subsequent step involves sophisticated target selection, utilizing computational platforms to prioritize ideal targets with high tumor specificity and homogeneous expression. Perhaps most critically, future efforts must focus on identifying and validating predictive biomarkers that can forecast response and resistance, including factors such as tumor mutational burden, the composition of the tumor microenvironment, and on-target antigen density. Without such a structured framework and predictive tools, the risk of patient selection failure and trial attrition remains high, hindering the successful integration of this powerful technology into the clinical management of NENs.

Continued fundamental and clinical research is essential to realize the full potential of CAR-T therapy and achieve the primary goal of improving the prognosis and quality of life for patients with resistant and metastatic NEN.
